# All-in-one medical image-to-image translation

**DOI:** 10.1016/j.crmeth.2025.101138

**Published:** 2025-08-11

**Authors:** Luyi Han, Tao Tan, Yunzhi Huang, Haoran Dou, Tianyu Zhang, Yuan Gao, Xin Wang, Chunyao Lu, Xinglong Liang, Yue Sun, Jonas Teuwen, S. Kevin Zhou, Ritse Mann

**Affiliations:** 1 Department of Radiology and Nuclear Medicine, Radboud University Medical Centre, 6525 GA Nijmegen, the Netherlands; 2 Department of Radiology, Netherlands Cancer Institute, 1066 CX Amsterdam, the Netherlands; 3 Faculty of Applied Sciences, Macao Polytechnic University, Macao 999078, China; 4 Jiangsu Key Laboratory of Intelligent Medical Image Computing, School of Artificial Intelligence, Nanjing University of Information Science and Technology, Nanjing 210044, China; 5 School of Computing, University of Leeds, Leeds LS2 9JT, UK; 6 GROW School for Oncology and Developmental Biology, Maastricht University Medical Centre, 6202 AZ Maastricht, the Netherlands; 7 Department of Radiation Oncology, Netherlands Cancer Institute, Plesmanlaan 121, 1066 CX Amsterdam, the Netherlands; 8 School of Biomedical Engineering & Suzhou Institute for Advanced Research, University of Science and Technology of China, Suzhou 215123, China; 9 Key Lab of Intelligent Information Processing of Chinese Academy of Sciences (CAS), Institute of Computing Technology, CAS, Beijing 100190, China

**Keywords:** multi-domain medical image, image-to-image translation, contrastive language-image pre-training, representation learning, zero-shot domain adaptation

## Abstract

The growing availability of public multi-domain medical image datasets enables training omnipotent image-to-image (I2I) translation models. However, integrating diverse protocols poses challenges in domain encoding and scalability. Therefore, we propose the “every domain all at once” I2I (EVA-I2I) translation model using DICOM-tag-informed contrastive language-image pre-training (DCLIP). DCLIP maps natural language scan descriptions into a common latent space, offering richer representations than traditional one-hot encoding. We develop the model using seven public datasets with 27,950 scans (3D volumes) for the brain, breast, abdomen, and pelvis. Experimental results show that our EVA-I2I can synthesize every seen domain at once with a single training session and achieve excellent image quality on different I2I translation tasks. Results for downstream applications (e.g., registration, classification, and segmentation) demonstrate that EVA-I2I can be directly applied to domain adaptation on external datasets without fine-tuning and that it also enables the potential for zero-shot domain adaptation for never-before-seen domains.

## Highlights

• EVA-I2I unifies multi-domain medical I2I translation in a single model
• DCLIP prompt encoding enables semantic domain control and scalability
• Outperforms baselines in I2I translation quality and zero-shot domain adaptation
• Enables direct deployment in clinical tasks without fine-tuning

## Motivation

The rise of large-scale multi-domain medical imaging datasets has motivated the development of generalizable image-to-image (I2I) translation models. A core challenge lies in effectively encoding diverse imaging protocols while maintaining semantic consistency and adaptability across domains. Traditional multi-domain I2I approaches often rely on task-specific architectures or discrete domain labels, limiting their scalability and generalization. Inspired by advances in contrastive language-image pre-training and prompt-driven generation, we introduce a unified and extensible framework that leverages DICOM-informed semantic representations to perform I2I translation across multiple medical imaging domains simultaneously.

## Graphical abstract

**Figure:**
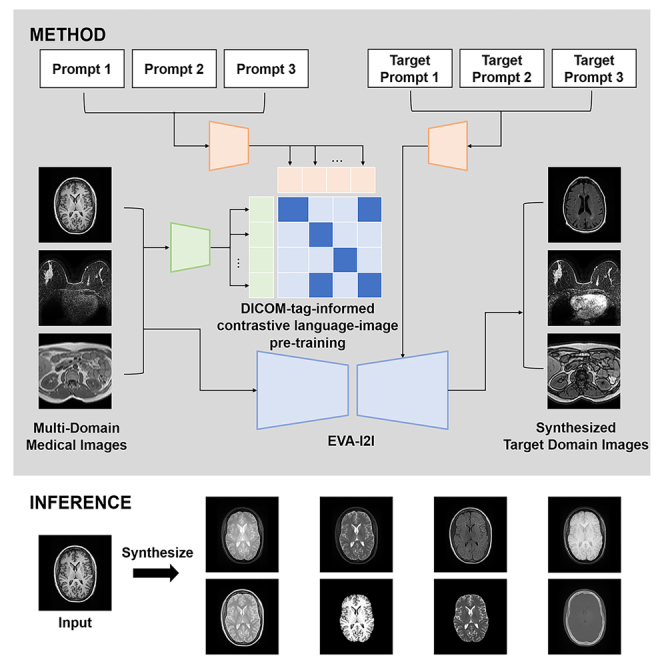

## Teaser

Han et al. propose EVA-I2I, a scalable image-to-image translation framework that synthesizes medical images across diverse domains using DICOM-tag-informed language-image pre-training. This method enables generalization to unseen domains and direct clinical application, advancing robust AI integration in medical imaging.

## Introduction

The application of deep learning in medical image analysis has gained popularity, but addressing the issue of model generalization remains crucial to its practical use in clinical settings.^1^ One of the reasons is the domain shift between datasets,^2^ i.e., the training set (source domain) and the test set (target domain) are in different distributions,^3^ often due to data being collected from different centers with varying scanning parameters or even different modalities.^4^ Many studies^5^^,^^6^^,^^7^ have focused on combating the domain shift in medical image analysis by implementing domain adaptation (DA) or data harmonization due to the lack of a large number of annotated training data that can cover the test set distribution.

Image-to-image (I2I) translation can be used for DA that directly aligns images by converting them from the source domain to the target domain.^4^ This technique has been widely used in the DA of medical images. One of its benefits is that it decouples DA from downstream models, making it applicable to various downstream tasks, such as registration,^8^ classification,^9^ and segmentation.^10^ It is worth noting that some I2I works have shown promising results, but few have conducted external validation studies^11^ due to the cynical performance dropping when migrating I2I. Additionally, in clinical settings, small sample sizes and missing sequences can complicate matters and result in poor results when directly training I2I on the target domains.^4^

**Figure 1:**
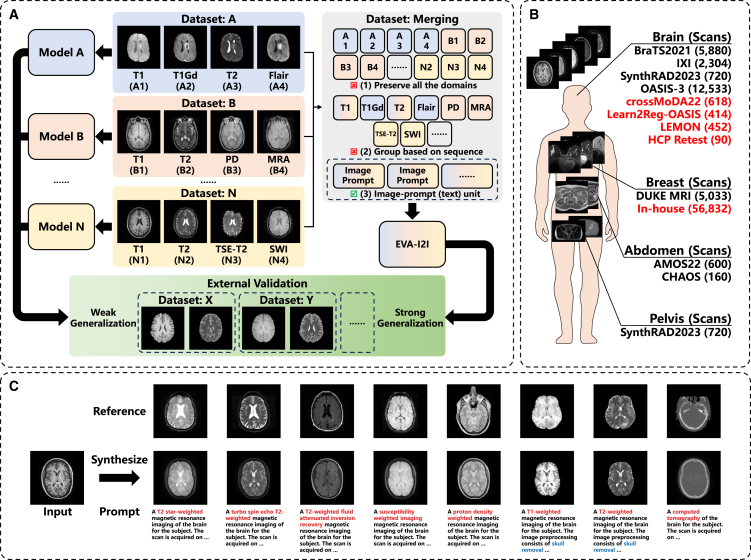
Overview of the proposed EVA-I2I model (A) Integrating domains from different datasets allows training an I2I translation model with strong generalization. (B) Datasets used in this work, where datasets for external validation are in red. (C) The proposed EVA-I2I model transforms the input image to different domains, given prompts. Note that the reference image is an example of the given prompt and is not used for inference.

Because the number of multi-domain public datasets increases, it now becomes possible to develop an omnipotent multi-domain I2I translation model. The large number of learned domains can cover more target domains, increase model generalization capabilities, and facilitate the acquisition of shared representation of anatomical structures across various domains.^4^ However, as shown in Figure 1A, due to the diverse scanning devices and subtle differences in parameter settings used, integrating datasets and constructing multi-domain I2I translation models can be challenging in encoding different domains while preserving semantic relationships and ensuring scalability. Existing studies partition data into a set of domains (e.g., based on modality or sequence), and it is typical to use one-hot encoding to differentiate between the various domains. Han et al.^12^ organize BraTS2021^13^ by placing the T1 (T1-weighted), T1Gd (contrast-enhanced T1), T2 (T2-weighted), and Flair (fluid-attenuated inversion recovery) sequences in a specific order and use one-hot encoding to represent the output sequences, such as “0100” for outputting T1Gd. Some works^14^^,^^15^ consider enriching the representation by additionally merging the encoding of the input sequence. One-hot encoding has some quite apparent limitations. Expanding the encoded sequence length to accommodate domain expansion can be quite challenging. Moreover, one-hot encoding cannot distinguish semantic differences across multiple domains, such as capturing the semantic relationship among A3, B2, and N2 in Figure 1A. Although all three are T2 images, the semantic distance between A3 (removed skull) and others (with skull) should be larger than B2 and N2.

Recent one-to-one methods^16^^,^^17^ have made progress in medical I2I but are difficult to apply to multi-domain scenarios due to the geometrical increase in the number of required models and training time as the number of domains grows. For natural images, ComboGAN^18^ sets up separate encoders, decoders, and discriminators for each domain, which achieves multi-domain I2I in one training process, but the number of models still grows linearly with the increasing number of domains. MUNIT^19^ disentangles images into separate style and texture components and then couples textures with different styles through adaptive instance normalization (AdaIN) to achieve multi-domain I2I. StarGAN^20^ introduces a multi-branch discriminator and a mapping net to enhance style encoding clustering. Disentangle-based methods can bring diverse synthesis results^10^ but have certain limitations, as they require the extraction of style encoding from the target domain during inference. For medical images, Sharma et al.^21^ propose a multi-channel input and output generator that produces various domains using distinct channels. This architecture avoids increasing the number of generators, but it may present challenges when expanding to new domains. Some studies^12^^,^^14^^,^^22^ use one-hot encoding to define the output domain. Chen et al.^14^ implement AdaIN to control the generator for synthesizing images of the desired domain, while Han et al.^12^ choose dynamic convolutions to achieve this. Referring to disentangle-based models, Jiang et al.^22^ combine one-hot coding with additional style encoding extracted from the images and use variational auto-encoder (VAE) to constrain the style encoding to match a Gaussian universal prior. Conditional models ensure flexible inference and scalability in modeling, but using one-hot encoding can be challenging in presenting subtle semantic relationships between domains. Therefore, we propose using prompts to define distinct domains while controlling the image synthesis process with contrastive language-image pre-training (CLIP) encoding. Large-scale vision-language models, e.g., CLIP, present widespread success in semantic understanding^23^^,^^24^ and have been applied to medical image analysis. Several studies^25^^,^^26^ have explored the correlation between chest X-ray (CXR) and its corresponding medical report, attaining zero-shot classification capabilities with image-report contrastive learning. Qin et al.^27^ create medical prompts to guide the vision-language model for object recognition and detection, showing success in zero/few-shot detection in the medical imaging field. Liu et al.^28^ propose using CLIP to help the model understand the relationship between different anatomical structures, resulting in the successful segmentation of desired targets from computed tomography (CT) images. Zhao et al.^29^ propose a joint-learning-based foundation model that determines different tasks through a textual description. For multi-domain I2I, CLIP has been incorporated with various vision generative models^30^^,^^31^^,^^32^ but rarely in the medical domain.^33^

In this work, we propose a virtual sequence with an “every domain all at once” I2I (EVA-I2I) translation model, which utilizes natural language to describe and identify different domains and uses DICOM-tag-informed CLIP (DCLIP) encoding to drive I2I synthesis. Specifically, we generate a corresponding prompt (text description) for a scan, which includes organ, modality, sequence, etc. We propose combining different datasets into one by pairing a scan and its prompt as the smallest unit, which can cluster domains and differentiate similar ones based on semantics. Encoding these prompts can guide the model to generate corresponding scans. Due to the characteristics of natural language, DCLIP encoding retains dense semantic relationships and is extensible. Compared to prior methods, our model offers three key advantages: (1) prompt-based representations allow flexible, scalable domain specification beyond fixed one-hot codes; (2) DCLIP enables aligned image-text embedding for semantically driven synthesis; and (3) the model supports translation across heterogeneous datasets without explicit domain labels. Additionally, as shown in Figure 1C, our method can learn and present all seen domains in the dataset simultaneously. Some of these domains have similar appearances, and subtle differences can be effectively distinguished through prompts and reflected in the generated images.

## Results

### Performance of in-domain and out-domain MRI I2I translation

As illustrated in Figure S1, our EVA-I2I consists of three main components: a text encoder, a structure encoder, and a conditional decoder. The text encoder embeds the medical prompts, which are composed of organ type, modality, sequence, view, etc., using DCLIP to produce a semantic representation aligned with the target domain. The structure encoder extracts anatomical features from the input image, which are finally rendered to the target image by the decoder according to the given domain representation. More details of the model components and training procedure are provided in the STAR Methods.

**Figure 2:**
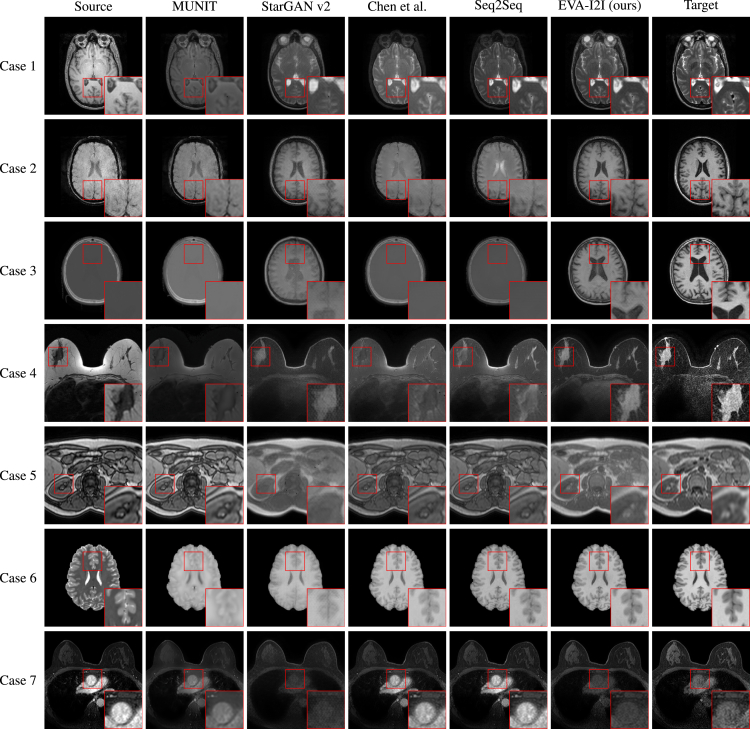
Axial visualization of translated images generated by comparison methods Cases 1–5 from the in-domain datasets show the transformation from T1 to T2, susceptibility weighted imaging (SWI) to T1, CT to T1, non-fat saturated to fat saturated, and out of phase to in phase. Case 6 and case 7 from the out-domain datasets show the transformation from T2 to T1 and post-contrast dynamic contrast-enhanced (DCE) to pre-contrast DCE. Our EVA-I2I attains better quality and a style that resembles the target image.

We compare the image translation performance of our EVA-I2I with that of other multi-domain I2I methods, such as MUNIT,^19^ StarGAN v.2,^20^ Chen et al.,^14^ and Seq2Seq.^12^ Table 1 illustrates the peak signal-noise rate (PSNR), structural similarity index measure (SSIM), and learned perceptual image patch similarity (LPIPS) of translated images generated by each method on the in- and out-domain datasets. All the comparison methods are trained on the training cohort of the in-domain datasets. Except for providing an input image when inferring, MUNIT and StarGAN v.2 need a reference image to extract style encoding from the target domain, and Chen et al., Seq2Seq, and the proposed EVA-I2I require an encoding (one hot or DCLIP) of the target domain to control the synthesis. Figure 2 shows the axial visualization of synthesized images generated by comparison methods. Results show that our EVA-I2I achieves significantly (p<0.05 for PNSR and LPIPS) better synthesis performance and more closely resembles the target style than other comparisons on both in- and out-domain datasets. To assess the computational efficiency of each method, we report the number of giga floating-point operations per second (GFLOPs) and the average inference time per slice in Table 1. Our method achieves moderate GFLOPs and inference times compared to other image translation models. Notably, the inference times of all compared methods are within a similar range and are negligible relative to the typical duration of multi-sequence MRI scanning (30–60 min). This suggests that our approach, along with the others, can be seamlessly integrated into clinical workflows without introducing much latency. More quantitative and visualization results are provided in Table S3 and Figures S2–S5. Furthermore, we evaluated the semantic consistency between the input and translated images using the MedSAM segmentation model.^34^ Specifically, MedSAM was applied independently to both the source and generated images, and the overlap between the resulting segmentation masks was measured. As shown in Figure S7, the images generated by our method yield the highest segmentation consistency with the input images, indicating that our approach preserves structural information and achieves strong semantic alignment across domains.

**Table 1:** The quantitative results of image translation for different comparisons on in- and out-domain datasets

Method	In-domain datasets (testing cohort)			Out-domain datasets			GFLOPS	Infer time (s)
	PSNR (dB) ↑	SSIM ↑	LPIPS ↓	PSNR (dB) ↑	SSIM ↑	LPIPS ↓		
MUNIT^19^	22.0 ± 2.9	0.805 ± 0.102	28.1 ± 16.5	20.4 ± 2.8	0.880 ± 0.014	17.2 ± 2.2	152.1	0.212
StarGAN v.2^20^	22.5 ± 2.9	0.793 ± 0.125	32.0 ± 16.6	21.9 ± 2.2	0.887 ± 0.014	19.7 ± 1.8	90.7	0.075
Chen et al.^14^	20.8 ± 3.4	0.799 ± 0.096	29.8 ± 17.1	23.3 ± 1.5	0.930 ± 0.017	10.7 ± 1.7	266.5	0.062
Seq2Seq^12^	21.8 ± 4.2	0.806 ± 0.136	26.2 ± 19.1	22.7 ± 2.4	0.899 ± 0.039	10.7 ± 1.9	296.4	0.078
EVA-I2I (ours)	**23.6** ± **3.6**	**0.807** ± **0.151**	**21.1** ± **15.7**	**24.3** ± **1.7**	**0.931** ± **0.017**	**9.54** ± **1.71**	214.6	0.109

The best results are in bold.

Table 2 illustrates the quantitative results of the ablation study on domain encoding, model backbone, and training loss function. We compare three domain encodings to equip our EVA-I2I, including one hot,^12^ BioBERT,^35^ and DCLIP (proposed). BioBERT encoding can extract semantic information from medical prompts, leading to better synthesis results than one-hot encoding, and our proposed DCLIP encoding aligns text and images, making its synthesis performance significantly (p<0.05 for PNSR and LPIPS) better than BioBERT encoding, which cannot align text and images. We also compare three model backbones of the structure encoder $E_{\Phi }$ in EVA-I2I, including ResNet,^36^ Swin-Transformer,^37^ and ConvNeXt (proposed). The ConvNeXt backbone slightly outperforms other methods. Moreover, we also evaluate the effectiveness of cross-dataset collaborative learning. Results show that the model trained with $L_{\text{fine}}$ significantly (p<0.05 for PNSR and LPIPS) outperforms the model only trained with $L_{\text{init}}$.

**Table 2:** The quantitative results of ablation study for EVA-I2I on domain encoding, model backbone, and training loss function

Ablation	Method	In-domain datasets (testing cohort)			Out-domain datasets		
		PSNR (dB) ↑	SSIM ↑	LPIPS ↓	PSNR (dB) ↑	SSIM ↑	LPIPS ↓
Encoding	one hot	21.9 ± 4.0	0.806 ± 0.139	26.2 ± 18.9	22.8 ± 2.3	0.901 ± 0.028	10.7 ± 1.9
	BioBERT	22.2 ± 2.9	0.806 ± 0.121	24.7 ± 17.5	23.5 ± 1.8	0.920 ± 0.019	10.4 ± 1.7
	DCLIP (ours)	**23.6** ± **3.6**	**0.807** ± **0.151**	**21.1** ± **15.7**	**24.3** ± **1.7**	**0.931** ± **0.017**	**9.54** ± **1.71**
Backbone	ResNet	23.4 ± 3.6	0.807 ± 0.152	21.3 ± 15.9	24.1 ± 1.7	0.930 ± 0.017	9.86 ± 1.73
	Swin-Transformer	23.5 ± 3.6	0.806 ± 0.151	21.3 ± 15.9	24.3 ± 1.7	0.929 ± 0.017	9.80 ± 1.73
	ConvNeXt (ours)	**23.6** ± **3.6**	**0.807** ± **0.151**	**21.1** ± **15.7**	**24.3** ± **1.7**	**0.931** ± **0.017**	**9.54** ± **1.71**
Loss	EVA-I2I-$L_{\text{init}}$	23.2 ± 3.7	0.805 ± 0.153	22.8 ± 16.4	23.8 ± 1.8	0.926 ± 0.016	10.2 ± 1.7
	EVA-I2I-$L_{\text{fine}}$	**23.6** ± **3.6**	**0.807** ± **0.151**	**21.1** ± **15.7**	**24.3** ± **1.7**	**0.931** ± **0.017**	**9.54** ± **1.71**

The best results are in bold.

### Training with multiple datasets increases model generalization

We compare the generalization performance of models trained on a single dataset and models trained on multiple datasets. For the single-dataset training experiments, we train Pix2Pix^38^ for T1-T2 brain MRI translation tasks on BraTS2021, IXI, and OASIS-3, respectively, and then directly test the model on LEMON and HCP Retest datasets. For our proposed EVA-I2I and other multi-domain I2I translation models, we utilize multiple in-domain datasets for training. Tables S4 and S5 show that Pix2Pix trained with BraTS2021 achieves better results than those trained with IXI and OASIS-3 because BraTS2021 has brain MRI with skull removal, which is the same in the LEMON and HCP Retest datasets. The compared multi-domain I2I translation methods are inferior to Pix2Pix because they fail to manage the domain semantics between multiple datasets. In contrast, our method can capture the subtle differences between the semantics of different domains, resulting in better generalization performance than Pix2Pix.

### Fine-grained control of synthesis images given by the prompt

**Figure 3:**
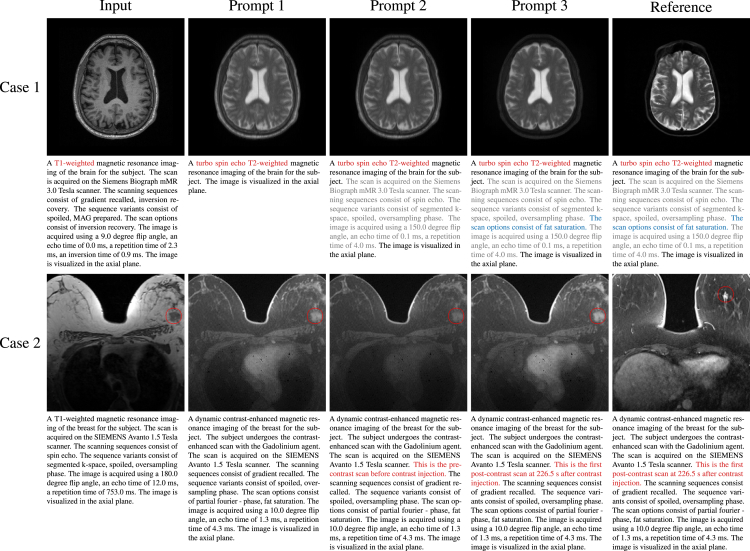
Axial visualization of the impact of changing prompts on generated images In case 1, altering the sequence (prompt 1) results in a noticeable change in the appearance of the image. Including additional scan information in prompt 2 has a minimal impact on the appearance. Fat saturation (prompt 3) further suppresses the intensity of fat tissue under the skin, thus rendering the generated image more consistent with the reference image. In case 2, when the specific time point of DCE-MRI is not specified (prompt 1), the intensity enhancement of the cardiac and tumor (red circle) is between the time points before contrast (prompt 2) and the first post-contrast (prompt 3). Note that the proposed model requires only the input image and the target prompt. The reference image is not used as an input but is provided solely for comparison. It corresponds to the given prompt but is from a different subject because the input case lacks the corresponding image of the reference prompt.

Text offers better control over details than one-hot encoding. As shown in Figure 3, modifying the modality or MRI sequence in the prompt can bring the most intuitive changes to the image appearance, followed by some imaging techniques, such as fat saturation, that will bring subtle changes in the image appearance. Other information, such as devices and scanning parameters, has little impact on the appearance. This may be because the latter causes smaller appearance changes than the former, and relevant information is often missing from the training data. Furthermore, the description of the time points can control the intensity enhancement produced by the contrast agent in DCE-MRI. If time-point descriptions are missing, the model tends to generate average intensities across multiple time points. Our EVA-I2I can control subtle changes in the generated image by altering the input prompt, which is not achievable by other methods unless using well-designed one-hot encoding to encode each keyword.

### Translate images from never-before-seen domains

We further explore the potential of zero-shot I2I translation using our EVA-I2I, i.e., translating images from never-before-seen domains to a seen domain. Figure S6 shows the translation from wash-in (subtraction between post- and pre-contrast DCE) to T1 images of the in-house dataset and from T1Gd (with fat saturation and remaining skulls) to T2 images of the crossMoDA22 dataset. The wash-in and T1Gd images are never-before-seen domains in the in-domain datasets. StarGAN v.2 outperforms other comparison methods because it acquires style information from the target domain. Our EVA-I2I surpasses StarGAN v.2 in terms of structure details, and it does not require style encoding, only medical prompts.

### Visualization of prompt embedding and prompt-based zero-shot classification

**Figure 4:**
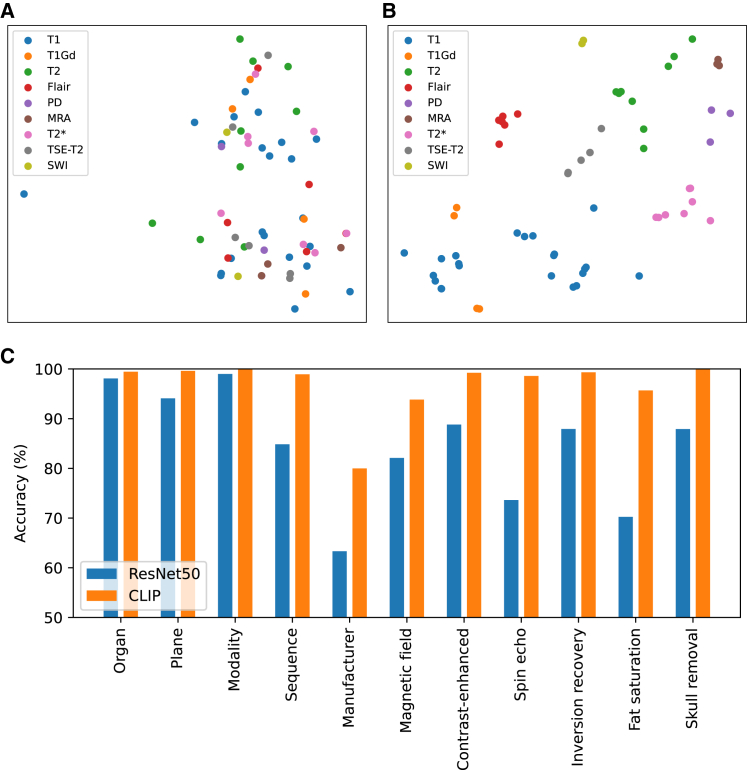
Visualization of prompt embedding (A) The t-SNE visualization of prompt embedding space of BioBERT encoding. (B) The t-SNE visualization of prompt embedding space of DCLIP encoding. (C) Bar chart of zero-shot classification on medical imaging characters.

We show the t-distributed stochastic neighbor embedding (t-SNE) visualization of prompt embedding space on different brain MRI sequences for BioBERT^35^ encoding and DCLIP encoding in Figure 4. DCLIP encoding highlights a more cohesive feature cluster, effectively conveying domain semantics. Specifically, for DCLIP encoding, T1 and T1Gd (T1 after contrast injection) are closer in the prompt embedding space and far from other sequences. In contrast, BioBERT does not maintain a clear separation between different sequences in the embedding space. It is shown that DCLIP-based encoding enables the model to capture complex domain relationships and learn sophisticated and structured feature embedding.

DCLIP-based encoding can effectively characterize prompt embeddings and also help establish a relationship between prompt and image. To support this, we experiment to evaluate the zero-shot classification ability of DCLIP encoding on the in-domain datasets. Specifically, we use images to match their most relevant prompts, i.e., the item with the largest cosine similarity, to classify some image characteristics, e.g., organ, the plane of view, modality, sequence, manufacturer, etc. As a comparison, we train a ResNet50^36^ model using these given characteristics as labels in a supervised pattern. Figure 4 shows the image classification results. Without additional annotations, the classification performance of DCLIP encoding has exceeded the supervised learning-based method.

### Downstream clinical tasks: Brain MRI registration

**Figure 5:**
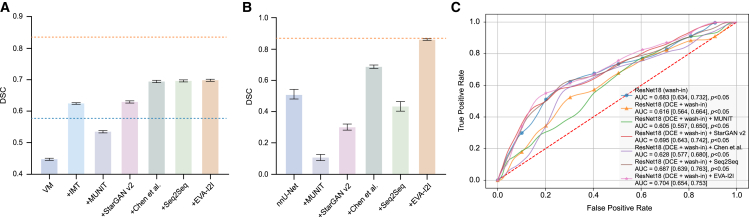
Performance of external domain adaptation (A) The bar chart of brain MRI registration on the HCP Retest dataset. (B) The bar chart of vestibular schwannoma segmentation on the crossMoDA22 dataset. (C) The receiver operating characteristic (ROC) curve of the neoadjuvant therapy (NAT) response prediction on the in-house dataset. Data are represented as mean ± SEM.

As shown in Figure 5A and Table S6, we utilized out-domain datasets to build cross-modality brain MRI registration experiments. The Dice coefficient similarity (DSC) for VoxelMorph (VM) is improved from 0.574 on Rigid to 0.837 on Learn2Reg-OASIS but drops a lot more on HCP Retest and LEMON datasets than that of Learn2Reg-OASIS (0.837) due to never-before-seen domains during training. Transforming T2 to T1 by DA methods can resolve the issue, but this is challenging without training on the source and target domain datasets. We compare different DA methods with the proposed EVA-I2I. Except for image modality translation (IMT)^8^ (with fine-tuning), all the DA methods are trained on the in-domain datasets and directly apply T2-to-T1 transformation on the HCP Retest and LEMON datasets. Table S6 shows that EVA-I2I achieves a better DSC than other DA methods.

### Downstream clinical tasks: Vestibular schwannoma segmentation

Figure 5B and Table S7 illustrate the external DA performance of our method on vestibular schwannoma segmentation. Directly applying the segmentation model to another domain leads to impairment of the segmentation performance. The results show that the proposed EVA-I2I extremely enhances the segmentation performance of nnU-Net, whose improvement outperforms other DA methods, showing the potential of zero-shot DA. The segmentation performance of the DA methods for small vestibular schwannoma (VS) is hard to compare with MSF-Net because DA methods are trained with an image spacing of 1×1 × 1 mm, while MSF-Net is trained with an image spacing of 0.412×0.412 × 1 mm, showing better VS structure. This is a limitation of EVA-I2I: it is trained with fixed image spacing and cannot adapt to tasks needing smaller image spacing.

### Downstream clinical tasks: Breast cancer pCR early prediction

Figure 5C shows the external DA performance of our method on breast cancer pathological complete response (pCR) early prediction. The performance of ResNet18 (DCE + wash-in) is severely degraded compared to ResNet18 (wash-in), which makes sense because non-fat-saturated (FS) DCEs have more noticeable differences in appearance than FS DCEs. After employing different DA methods, the predicted area under the ROC curve (AUC) has improved. The proposed EVA-I2I achieves the most apparent improvement with AUC and exceeds the performance of ResNet18 (wash-in) by leveraging breast tissue information in DCEs.

## Discussion

In this work, we introduce medical prompts to define and identify medical images in distinct domains and further propose EVA-I2I to achieve multi-domain I2I translation via the guide of DCLIP encoding of text descriptions of target domains. Medical prompts have advantages over one-hot encoding: (1) they can integrate multiple datasets and avoid conflicts between different domains and (2) they contain rich semantic information, which can effectively differentiate between inner-sequence and inter-sequence imaging differences. Table S4 illustrates that training models with multiple datasets may not always enhance synthesis performance. One-hot encoding fails to address domain conflicts that may arise when combining diverse datasets, while using medical prompts has been proven to address this issue and result in improved synthesis performance compared to using a single dataset. As shown in Figure 3, adding the keyword “fat saturation” and a description of the time point in the prompt can distinguish Turbo Spin Echo (TSE)-T2 with different appearances and DCE-MRI with different intensity enhancement of tumors, respectively. Achieving equivalent capabilities with one-hot encoding requires a more intricate design and is hard to broaden. Moreover, we propose using DCLIP for image-text alignment rather than encoding text with an unaligned text encoder such as BioBERT. Table 2 demonstrates that our EVA-I2I, guided by DCLIP encoding, produces better synthesis results than the one guided by BioBERT encoding. This is because DCLIP encoding can more effectively capture the semantic relationship between domains after aligning text and image. As shown in Figure 4, this semantic relationship is related to the difference in image appearance and cannot be represented by BioBERT encoding. Furthermore, the proposed cross-dataset collaborative learning approach can establish a connection between distinct datasets and achieve I2I translation across datasets. This effectively broadens the range of target domains that our model can generate, as shown in Figure 1, and improves the synthesis performance, as presented in Table 2.

Our EVA-I2I demonstrates strong generalization performance with the help of multi-dataset training and the guidance of DCLIP encoding for medical prompts. Table 1 and Figure 2 illustrate that the proposed model can achieve better synthesis performance on external validation than other comparisons. Although it may seem counterintuitive that performance on some out-domain datasets surpasses that on in-domain datasets, this can be explained by differences in organ type and synthesis difficulty. For example, HCP (out-domain) contains skull-stripped brain MRIs that resemble BraTS2021 (in-domain), leading to comparable results. In contrast, CHAOS (in-domain) involves abdominal MRIs with greater anatomical complexity, resulting in lower synthesis quality. As a result, the average in-domain metrics may appear lower, but method comparisons within each dataset remain consistent and valid. Figure S6 further demonstrates the zero-shot I2I translation capability of the proposed model, which can translate never-before-seen domains in the training datasets into seen domains. It dramatically expands the scope of application for our EVA-I2I to include tasks that cannot be accomplished by the compared models. Moreover, the generalization of our EVA-I2I is also reflected in downstream applications, which is also the purpose of applying DA in clinical practice. Figure 5 and Tables S6 and S7 show the DA performance of our model for cross-modality registration, classification, and segmentation on external datasets, respectively. Many studies on DA have shown that in order to achieve good results, the model needs to be fine-tuned on the target domain. However, our approach does not require fine-tuning and can directly yield good results on external validation datasets. Even in the case of zero-shot DA, our method can achieve performance that is comparable to the fine-tuned approach.

In this paper, we propose a DCLIP-driven model for medical I2I translation. We integrate DCLIP encoding into a conditional synthesis model to establish semantic relationships between the description and appearance of multi-domain images, resulting in a flexible and powerful model. The model effectively learns from diverse public datasets and identifies shared information between medical images, achieving synthesis accuracy surpassing other DA methods on in- and out-domain datasets. Moreover, downstream applications demonstrate that the EVA-I2I can be utilized for DA on external datasets without fine-tuning, highlighting its potential to enhance the generalization of existing deep learning models.

### Limitations of the study

This study also has some limitations. First, the proposed model has a fixed image resolution for training, limiting its ability to perform well in higher-resolution situations when performing downstream applications. Moreover, our model is only capable of processing 2D slices. Although we have incorporated the plane of view in the medical prompts to enable our model to make inferences from different views, it still struggles to comprehend complex 3D structures efficiently, which may lead to inter-slice discontinuities, particularly during zero-shot I2I translation tasks. Furthermore, the out-domain datasets do not cover the same organs or imaging domains as the in-domain datasets, which may lead to inconsistencies and hinder direct comparability of performance metrics between the in-domain and out-domain datasets.

## Resource availability

### Lead contact

Requests for further information and resources should be directed to and will be fulfilled by the lead contact, Tao Tan (taotan@mpu.edu.mo).

### Materials availability

This study did not generate new unique materials or reagents.

### Data and code availability

• This paper analyzes existing, publicly available data. Accession numbers or web links for the public available datasets are listed in the key resources table.
• All source codes are publicly available at https://github.com/fiy2W/eva_i2i. An archival DOI is listed in the key resources table.
• Any additional information required to reanalyze the data reported in this paper is available from the lead contact upon request.

## Acknowledgments

L.H. was funded by a Chinese Scholarship Council (CSC) scholarship (no. 202006240065). This work is supported by 10.13039/501100006469Science and Technology Development Fund of Macao (0105/2022/A) and a Macao Polytechnic University grant (RP/FCA-08/2024). The authors would like to acknowledge the Research High Performance Computing (RHPC) facility of the Netherlands Cancer Institute (NKI).

## Author contributions

Conceptualization, L.H., T.T., and R.M.; methodology, L.H., Y.H., H.D., and T.T.; data curation, H.L., X.W., Y.G., and T.Z.; formal analysis, L.H., X.W., Y.G., T.Z., H.D., Y.H., C.L., X.L., and R.M.; software, J.T.; project administration, R.M. and T.T.; resources, R.M. and T.T.; writing – original draft, L.H.; writing – review & editing, T.T., Y.S., and S.K.Z.; supervision, T.T. and R.M.

## Declaration of interests

The authors declare no competing interests.

## STAR★Methods

### Key resources table

**Table:** 

REAGENT or RESOURCE	SOURCE	IDENTIFIER
**Deposited data**		

AMOS22 dataset	Multi-Modality Abdominal Multi-Organ Segmentation Challenge 2022	https://amos22.grand-challenge.org/
BraTS2021 dataset	Brain Tumor Segmentation Challenge 2021	http://www.braintumorsegmentation.org/
CHAOS dataset	Combined (CT-MR) Healthy Abdominal Organ Segmentation Challenge	https://chaos.grand-challenge.org/
Duke Breast Cancer MRI dataset	The Cancer Imaging Archive	https://www.cancerimagingarchive.net/collection/duke-breast-cancer-mri/
IXI dataset	Human Connectome Project	https://brain-development.org/ixi-dataset/
SynthRAD2023 dataset	Synthesizing computed tomography for radiotherapy challenge	https://synthrad2023.grand-challenge.org
OASIS-3 dataset	Open Access Series of Imaging Studies	https://www.oasis-brains.org/
crossMoDA22 dataset	Cross-Modality Domain Adaptation Challenge	https://crossmoda2022.grand-challenge.org/
Learn2Reg-OASIS dataset	Learn2Reg Challenge	https://learn2reg.grand-challenge.org
LEMON dataset	Max Planck Institut Leipzig Mind-Brain-Body Dataset	https://fcon_1000.projects.nitrc.org/indi/retro/MPI_LEMON.html
HCP Retest dataset	Human Connectome Project	https://db.humanconnectome.org/data/projects/HCP_Retest

**Software and algorithms**		

EVA-I2I	This paper	https://github.com/fiy2W/eva_i2i and https://doi.org/10.5281/zenodo.15815446
pytorch 2.0.0	PyTorch	https://pytorch.org
torchvision 0.16.2	PyTorch	https://pytorch.org
huggingface-hub 0.27.0	Hugging Face	https://huggingface.co
tokenizers 0.15.2	Hugging Face	https://huggingface.co/docs/tokenizers/index
transformers 4.39.3	Hugging Face	https://huggingface.co/docs/transformers/index
numpy 1.26.3	NumPy	https://numpy.org/
scipy 1.11.4	SciPy	https://scipy.org/
scikit-learn 1.4.1	scikit-learn	https://scikit-learn.org/stable/
scikit-image 0.22.0	scikit-image	https://scikit-image.org/
SimpleITK 2.3.1	SimpleITK	https://simpleitk.org/
timm 0.9.12	timm	https://github.com/huggingface/pytorch-image-models
lpips 0.1.4	LPIPS	https://github.com/richzhang/PerceptualSimilarity

### Experimental model and study participant details

#### Data collection

Table S1 illustrates the detailed information of datasets used in this work, divided into in- and out-domain datasets. In-domain datasets (D01-D07) comprise 7 public datasets with 27,950 brain, breast, abdomen, or pelvis scans (3D volumes). They are randomly split into training, validation, and independent testing cohorts with a ratio of 3:1:1 based on patients. Out-domain datasets include 4 public datasets (D08-D11) and an in-house dataset (D12) aiming for extra external validation and downstream applications. Within each sub-dataset, multi-domain cases are pre-aligned. In contrast, alignment across different datasets is not required. The in-house dataset includes 2,101 breast cancer patients treated with neoadjuvant therapy (NAT) between 2000 and 2020 at the Netherlands Cancer Institute (NKI) in Amsterdam, the Netherlands. This retrospective study was approved by the Institutional Review Board of the NKI, with registration number IRBd21-059. All data collection and usage were conducted in compliance with institutional ethical guidelines. The in-house cohort consisted exclusively of female patients, ranging in age from 19 to 82 years, with a median age of 50. The overall pathological complete response (pCR) rate in this cohort was 31%. Each patient has two to four multi-sequence MRI visits to acquire T1, T2, DCE, and diffusion-weighted imaging (DWI). Because of the long inclusion period, the MRI protocol for breast examination has changed, with variations in the scanning techniques used. Before 2015, scanning was acquired using 2D sequences in the coronal plane, while after 2015, it shifted toward 3D scanning in the transverse plane. Furthermore, in recent years, T1 and DCE have been scanned using fat-saturation techniques. DCE is acquired with 6 timepoints within 7.5 min after administering contrast. Additionally, DWI is performed using four different b values of 0, 150, 800, and 1,500. The MRI scans are obtained using Philips Ingenia 3.0-T scanners. We also generate the corresponding skull-removed images for brain scans of the in-domain datasets to increase the number of domains. The downstream tasks delineated in this paper are exclusively related to MRI, thus, CT datasets are not considered a part of the out-domain datasets. Images for the brain and breast are resampled to 1×1 × 1 mm, and images of the abdomen and pelvis are resampled to 2.5×1 × 1 mm.

### Method details

#### Medical prompt for domain description

With available information from scans, we design a template to build medical prompts (e.g., “A T2-weighted magnetic resonance imaging of the brain for the subject …”), which can describe and identify the corresponding scan. Table S2 illustrates the tags and keywords we extract from DICOM files. For datasets in which DICOM files are not provided, we manually collect relevant information from the summary Excel files provided by the corresponding public dataset. Besides these keywords from DICOM, we additionally define [Organ] token for organ (e.g., brain, breast, abdomen, and pelvis), [Modality] token for modality (e.g., magnetic resonance imaging, computed tomography, and cone beam computed tomography), [Sequence] token for MRI sequence (e.g., T1-weighted, T2-weighted, T2 star-weighted, and T2-weighted-fluid-attenuated inversion recovery), [SequenceFrameNumber] token for the numbers of the multi-timepoint sequences (e.g., first, second, and third), [Contrast] token for contrast agent (e.g., Gadolinium), [Preprocess] token for image preprocess (e.g., skull removal), and [Plane] token for the plane of view (e.g., axial, coronal, and sagittal) with the help of radiologists. We generate the medical prompt automatically based on the above keywords to describe the corresponding scan. Referred from,^28^ we define the medical prompt template for MRI as follows,

“A [Sequence] [Modality] of [Organ] for the subject. The subject undergoes the contrast-enhanced scan with the [Contrast] agent. The scan is acquired on the [Manufacter] [ManufacturerModelName] [MagneticFieldStrength] Tesla scanner. The scanning sequences consist of [ScanningSequence]. The sequence variants consist of [SequenceVariant]. The scan options consist of [ScanOptions]. The image is acquired using a [FlipAngle] degree flip angle, an echo time of [EchoTime] ms, a repetition time of [RepetitionTime] ms, an inversion time of [InversionTime] ms. The image preprocessing consists of [Preprocess]. The image is visualized in the [Plane] plane.”

The medical prompt template will be slightly adjusted when some keywords are unavailable. Prompt augmentation can enhance generalization by reducing reliance on fixed templates, and strengthen contextual understanding and language flexibility. We randomly delete some sentences (but preserve essential sentences extremely influencing the image style, e.g., involving information on organ, modality, and sequence) during the training process to achieve data augmentation. We initialize a prompt as a list of sentence dictionaries, which includes the content of the sentence and the mask of the sentence. If the sentence is essential, the mask is set to 1. During augmentation, the essential sentences are preserved, and others are randomly deleted. Moreover, a random shuffle is applied to the selected sentences in each training step.

#### DCLIP encoding with text and image encoders

To build the domain representation $\Theta_{s}$ with prompt, we apply DCLIP to encode the text because it can learn a well-aligned space between images and texts. As shown in Figure S1A, we directly adapt text encoder $E_{T}$ from CLIP^23^ and improve the image encoder $E_{I}$ with ConvNeXt^39^ backbone for better representation. We improve the pre-training referring to supervised contrastive learning^40^ because some prompts can correspond to multiple images, while the loss in^23^ cannot maximize the similarity between these samples.

The text encoder $E_{T}$ is a 12-layer Transformer with a width of 768 and 12 attention heads, adapted from CLIP.^23^ This transformer operates on a lower-cased byte pair encoding (BPE) representation of the text with a vocabulary size of 49,152. The maximum content length is limited to 256 to handle the long medical prompt. Furthermore, the text sequence is enveloped with [SOS] and [EOS] tokens, and the feature representation of the text is obtained by treating the activations of the highest layer of the transformer at the [EOS] token. The feature representation is then layer-normalized and linearly projected into the domain encoding space as the domain representation $\Theta_{s}$.

The image encoder $E_{I}$ is improved from the ResNet-based architecture of CLIP,^23^ whose backbone is replaced with ConvNeXt-T^39^, making it more efficient, scalable, and accurate. $E_{I}$ commences with a convolutional layer featuring a kernel size of 7×7, a stride of 1×1, and an output channel of 96. It is then followed by ConvNeXt-T, which comprises four stages exhibiting channel numbers of 96, 192, 384, and 768, block numbers of 3, 3, 9, and 3, and strides of 4, 2, 2, and 2, respectively. The global average pooling layer is substituted with an attention pooling mechanism, a single Transformer-style multi-head QKV attention layer with the image global average-pooled conditioned query.

Since different subjects may have performed the same scan, i.e., one prompt corresponds to multiple images, we improve the DCLIP loss $L_{\text{CLIP}}$ referring the supervised contrast loss^40^ so that it can reduce the distance between these samples. For image X and prompt P pairs from a mini-batch of the dataset D, we define $L_{\text{DCLIP}}$ as follows,

(Equation 1) $$L_{\text{DCLIP}}=-\sum_{i\in D}\left(\frac{1}{\left|M\left(i,\cdot \right)\right|}\sum_{m\in M\left(i,\cdot \right)}\log\frac{\exp\left(z_{T}^{\left(i\right)}\cdot z_{I}^{\left(m\right)}/\tau \right)}{\sum_{j\in D}\;\exp\left(z_{T}^{\left(i\right)}\cdot z_{I}^{\left(j\right)}/\tau \right)}+\frac{1}{\left|M\left(\cdot ,i\right)\right|}\sum_{m\in M\left(\cdot ,i\right)}\log\frac{\exp\left(z_{I}^{\left(i\right)}\cdot z_{T}^{\left(m\right)}/\tau \right)}{\sum_{j\in D}\;\exp\left(z_{I}^{\left(i\right)}\cdot z_{T}^{\left(j\right)}/\tau \right)}\right)$$

where $z_{I}$ and $z_{T}$ are projections of the image representation $E_{I}\left(X\right)$ and the text representation $E_{T}\left(P\right)$, M indicates the label of the confusion matrix whose value is 1 when the prompt matches the image, and 0 for the rest. Note that, one prompt could match multiple images. M(i,·) presents items with the label of 1 from the i th row of M, M(·,i) presents items with the label of 1 from the i th column of M, τ=0.07 refers to the scalar temperature parameter. To construct M, we compare each image’s original prompt with each candidate text prompt: a match is established if all sentences in the candidate prompt appear in the original prompt, and all necessary components of the original prompt are present in the candidate. This ensures robust matching even under text augmentation and partial paraphrasing.

#### DCLIP-driven I2I translation model

As shown in Figure S1B, the architecture of the proposed EVA-I2I consists of the text encoder $E_{T}$, a structure encoder $E_{\Phi }$, and a conditional decoder G. First, we use the weight-frozen $E_{T}$ to convert the prompt into a DCLIP encoding T, which is an estimation of $\Theta_{s}$. Then, using $E_{\Phi }$, we extract the approximate structure representation Φ of the input image. Finally, with G, we can render the structure representation of the image into the corresponding scan using the input DCLIP encoding as a control. $E_{\Phi }$ and G have ConvNeXt^39^ backbone equipped with self-attention blocks, and G is implemented with dynamic convolutions^12^ to embed T. Moreover, we develop a DCLIP-driven discriminator to simplify the training process of adversarial learning.

The structure encoder $E_{\Phi }$ starts with a convolutional layer with a kernel size of 7×7, a stride of 1×1, and an output channel of 96. Then it follows with a two-stage ConvNeXt backbone, exhibiting channel numbers of 192 and 384, block numbers of 1 and 3, and strides of 2 and 2, respectively. In addition, a self-attention block is inserted between each two ResNeXt blocks. The structure of the conditional decoder G is symmetrical to that of $E_{\Phi }$, except that the convolutional layers in the ResNeXt blocks are replaced by the HyperConv layers^12^ to receive the control of DCLIP encoding. The convolutional layer with a stride of 2 is replaced with a two-times upsampling layer followed by a convolutional layer with a stride of 1. The output of G is activated with a LeakyReLU layer.

Instead of defining a discriminator for each domain, we use a DCLIP-driven discriminator D, inspired by our generator, to handle all domains effectively. We employ ResNeXt blocks equipped with HyperConv layers^12^ in D. Specifically, D consists of three convolutional layers with a kernel size of 4, a stride of 2, and output channels of 96, 192, and 384, four conditional ResNeXt blocks inserted with self-attention blocks, and one convolutional layer with a kernel size of 1 and output channel of 1.

#### Inner-subject supervision

Figure S1C shows the training procedure of the proposed EVA-I2I. For paired samples from the same dataset, we can use supervised learning to constrain the synthesized images at the pixel and perceptual levels. The reconstruction loss for each paired source image $X_{\text{src}}$ and target image $X_{\text{tgt}}$ is as follows,

(Equation 2) $$L_{\text{rec}}=\lambda_{L1}\cdot \left({\left\|X_{\text{src}}^{\prime }-X_{\text{src}}\right\|}_{1}+{\left\|X_{\text{tgt}}^{\prime }-X_{\text{tgt}}\right\|}_{1}\right)+\lambda_{p}\cdot \left(L_{p}\left(X_{\text{src}}^{\prime },X_{\text{src}}\right)+L_{p}\left(X_{\text{tgt}}^{\prime },X_{\text{tgt}}\right)\right)\text{,}$$

where generated target image $X_{\text{tgt}}^{\prime }=G\left(E_{\Phi }\left(X_{\text{src}}\right)\|T_{\text{tgt}}\right)$, generated source image $X_{\text{src}}^{\prime }=G\left(E_{\Phi }\left(X_{\text{tgt}}\right)\|T_{\text{src}}\right)$, $T_{\text{src}}$ and $T_{\text{tgt}}$ are DCLIP encoding for prompts of $X_{\text{src}}$ and $X_{\text{tgt}}$, ${\left\|\cdot \right\|}_{1}$ refers to an $L_{1}$ loss, and $L_{p}$ indicates the perceptual loss based on pre-trained VGG19. $\lambda_{L1}$ and $\lambda_{p}$ are weight terms and experimentally set to be 10 and 0.1.

For paired samples, we also constrain their structure representation to be consistent, and the structure loss is,

(Equation 3) $$L_{\text{con}}={\left\|E_{\Phi }\left(X_{\text{src}}\right)-E_{\Phi }\left(X_{\text{tgt}}\right)\right\|}_{2}^{2}+L_{\text{PCL}}\left(E_{\Phi }\left(X_{\text{src}}\right),E_{\Phi }\left(X_{\text{tgt}}\right)\right)\text{,}$$

where $\|\cdot {\|}_{2}^{2}$ refers to a mean square error (MSE) loss, and $L_{\text{PCL}}$ indicates the contrastive loss at the pixel level. We propose using additional $L_{\text{PCL}}$ to restrict the consistency of image content, as MSE can lead to over-compression on the value range of features. For image $X_{\text{src}},X_{\text{tgt}}\in R^{1\times W\times H}$, $L_{\text{PCL}}$ is defined on the feature pixels located by common foreground mask $M_{0}=\left(X_{\text{src}}>0\right)\cap \left(X_{\text{tgt}}>0\right)$,

(Equation 4) $$L_{\text{PCL}}=-\sum_{i\in M}\log\frac{\exp\left(p_{i}\cdot q_{i}/\tau \right)}{\sum_{j\in M}\;\exp\left(p_{i}\cdot q_{j}/\tau \right)}\cdot \frac{\exp\left(q_{i}\cdot p_{i}/\tau \right)}{\sum_{j\in M}\;\exp\left(q_{i}\cdot p_{j}/\tau \right)}\text{,}$$

where $M\in Z^{1\times \frac{W}{4}\times \frac{H}{4}}$ is four times downsampled from $M_{0}\in Z^{1\times W\times H}$, $p,q\in R^{C\times \frac{W}{4}\times \frac{H}{4}}$ are normalization of the image content representations $E_{\Phi }\left(X_{\text{src}}\right)$ and $E_{\Phi }\left(X_{\text{tgt}}\right)$, i and j are pixel indices, $p_{i},q_{i},p_{j},q_{j}\in R^{C}$ are features corresponding to the pixels selected in M, and τ=0.07 refers to the scalar temperature parameter.

#### Cross-dataset collaborative learning

To maximize the utilization of multiple datasets, we propose constraints on unpaired samples between different datasets. Besides paired images $X_{\text{src}}$ and $X_{\text{tgt}}$, we randomly introduce an intermediate image $X_{\mathrm{int}}$ and its corresponding DCLIP encoding $T_{\mathrm{int}}$ from another subject in the mini-batch, and build a triangular consistency loss to establish mappings between domains across datasets. Note that, $X_{\mathrm{int}}$ is traversed from other datasets and for the same organ with $X_{\text{src}}$ and $X_{\text{tgt}}$.

(Equation 5) $$L_{\text{tri}}=\lambda_{L1}\cdot {\left\|X_{\mathrm{int}\to \text{tgt}}^{\prime }-X_{\text{tgt}}\right\|}_{1}+\lambda_{p}\cdot L_{p}\left(X_{\mathrm{int}\to \text{tgt}}^{\prime },X_{\text{tgt}}\right)\text{,}$$

where $X_{\mathrm{int}\to \text{tgt}}^{\prime }=G\left(E_{\Phi }\left(X_{\mathrm{int}}^{\prime }\right)\|T_{\text{tgt}}\right)$, and the generated intermediate image is $X_{\mathrm{int}}^{\prime }=G\left(E_{\Phi }\left(X_{\text{src}}\right)\|T_{\mathrm{int}}\right)$.

To make G to generate an image with an appearance similar to the intermediate image, we introduce adversarial loss and cosine similarity loss to constrain the image style and semantics, respectively.

(Equation 6) $$\min_{D}\;\max_{E_{\Phi },G}L_{\text{adv}}={\left\|D\left(X_{\mathrm{int}}\|T_{\mathrm{int}}\right)-1\right\|}_{2}^{2}+{\left\|D\left(X_{\mathrm{int}}^{\prime }\|T_{\mathrm{int}}\right)\right\|}_{2}^{2}\text{,}$$

(Equation 7) $$L_{\cos}=-\frac{\left\langle E_{I}\left(X_{\mathrm{int}}^{\prime }\right),T_{\mathrm{int}}\right\rangle }{\left\|E_{I}\left(X_{\mathrm{int}}^{\prime }\right)\right\|\cdot \left\|T_{\mathrm{int}}\right\|}\text{,}$$

where D is the DCLIP-driven discriminator, ⟨,⟩ refers to the inner product, and $E_{I}$ is frozen during training.

#### Total loss

We train the generator using only inner-subject supervision in the initial stage and additionally add cross-dataset collaborative learning in the fine-tuning stage. The total loss function in the initial stage and fine-tuning stage of the generative model can be summarized as,

(Equation 8) $$\begin{array}{cc} L_{\text{init}}= & L_{\text{rec}}+\lambda_{\text{con}}L_{\text{con}}\\ L_{\text{fine}}= & L_{\text{rec}}+\lambda_{\text{con}}L_{\text{con}}+L_{\text{tri}}+\lambda_{\text{adv}}L_{\text{adv}}+\lambda_{\cos}L_{\cos}\text{,} \end{array}$$

where we set $\lambda_{\text{con}}=1$, $\lambda_{\text{adv}}=5$, and $\lambda_{\cos}=0.1$ with experimental experience.

#### Training details

All the models are developed with PyTorch, and experiments are conducted on the NVIDIA Quadro RTX A6000 GPU. The EVA-I2I is trained using the AdamW optimizer with an initial learning rate of $10^{-4}$ and an input image size of 256×256. We select the best model by evaluating the best PSNR in validation cohorts of in-domain datasets. During training, images are first normalized with $N\left(X\right)=\left(\max\left(X,R_{\min}\right)-R_{\min}\right)/R_{X}$, where $R_{\min}$ is set to 0 for MRI and −1024 for CT and CBCT. $R_{X}$ is 99.5 percentile of X for MRI, 3000 for CT, and 1500 for CBCT, respectively. And then, the normalized images are padded or randomly cropped to 256×256×256. Finally, 2D slices are randomly selected in axial, coronal, or sagittal planes from the image volume. DCLIP is adopted with $L_{\text{DCLIP}}$ and a batch size of 60 for 50 epochs on the in-domain datasets. We initialize the proposed EVA-I2I by training 5 epochs on paired scans with $L_{\text{init}}$ and a batch size of 6. Then, we train the model with $L_{\text{fine}}$ and a batch size of 6 for 45 epochs on both paired and unpaired samples.

#### Downstream clinical tasks: Brain MRI registration

Cross-modality registration is widely used to align anatomical structures between different domains of medical images. Three public datasets from the out-domain datasets are utilized to build the brain image registration experiments. Learn2Reg-OASIS^41^ (T1-to-T1 mono-modality registration) is split into three cohorts, with 350 subjects for training the learning-based registration model (VoxelMorph^42^), 14 for validation, and 50 for testing. HCP Retest and LEMON (T2-to-T1 cross-modality registration) are utilized for external validation. All the deformation registration is based on rigid registration results. VM is only trained on the Learn2Reg-OASIS dataset and is directly tested on HCP Retest and LEMON datasets.

#### Downstream clinical tasks: Vestibular schwannoma segmentation

Cross-modality segmentation is a common clinical task that migrates knowledge to other domains, relieving the pressure of data annotation. We conduct the experiment on the crossMoDA22^43^^,^^44^ dataset for segmenting vestibular schwannoma (VS). Specifically, we train the segmentation model based on 227 annotated T1Gd (never-before-seen domain) images and test the model on another 96 unpaired T2 images. MSF-Net^45^ trains DA between the source and target domains as an upper bound for comparisons. We train the nnU-Net^46^ as a baseline model with annotated T1Gd images. We use different DA methods to transform T1Gd images into fake T2 images to re-train the nnU-Net. All the DA methods are trained with the in-domain datasets, which makes this task a zero-shot DA due to the never-before-seen T1Gd for VS in the training sets.

#### Downstream clinical tasks: Breast cancer pCR early prediction

Classification models have generalization problems when dealing with cross-domain or multi-center data. In the in-house dataset, the DCE protocol for breast examination has changed due to the long inclusion period. The early DCEs do not apply fat saturation (FS) techniques and are acquired using 2D sequences in the coronal plane. In contrast, the recent DCEs apply FS and shift toward 3D scanning in the transverse plane. This can lead to significant differences in image appearance. The wash-in image is a subtraction between post-contrast and pre-contrast DCE, which can highlight the tumor region and is not affected by FS but loses the context of breast tissue.^12^ Here, we conduct an experiment on the in-house dataset that predicts the response to neoadjuvant therapy (NAT) for breast cancer patients based on pre-NAT DCE-MRI. Specifically, we train a ResNet18^47^ model on the combination of FS DCEs and wash-in images from the in-house dataset and test the classification performance of the model on the combination of non-FS DCEs and corresponding wash-in images. We also train ResNet18 only on wash-in images as a benchmark for the prediction performance.

### Quantification and statistical analysis

#### Statistical analysis

Statistical analysis was done using Python 3.11.3. All statistical tests were conducted with a two-sided T-test, and p<0.05 indicated statistical significance. The assessment of translation performance is quantified through peak signal noise rate (PSNR), structural similarity index measure (SSIM), and learned perceptual image patch similarity (LPIPS^48^). Higher PSNR indicates better image fidelity, higher SSIM reflects better contrast and structural information, and lower LPIPS implies better human visual perception. Downstream applications involve registration, classification, and segmentation tasks. Dice coefficient similarity (DSC) is utilized to evaluate image registration quality, which measures spatial overlap between anatomical regions. The area under the ROC curve (AUC) is used to evaluate the classification performance. DSC and average symmetric surface distance (ASSD) are used to assess the segmentation performance on area and boundary overlapping, respectively. All calculation methods are as follows:

(Equation 9) $$\text{PSNR}=10\times \log_{10}\frac{R^{2}}{{\left\|X^{\prime }-X\right\|}_{2}^{2}}$$

where R=1 presents the image intensity range. $X^{\prime }$ is the synthesized image of comparison methods, and X is the ground truth.

(Equation 10) $$\text{SSIM}\left(x,y\right)=\frac{\left(2\mu_{x}\mu_{y}+c_{1}\right)\left(2\sigma_{xy}+c_{2}\right)}{\left(\mu_{x}^{2}+\mu_{y}^{2}+c_{1}\right)\left(\sigma_{x}^{2}+\sigma_{y}^{2}+c_{2}\right)}$$

where x and y are windows of $X^{\prime }$ and X, $\mu_{x}$ and $\mu_{y}$ are the pixel sample mean of x and y, $\sigma_{x}^{2}$ and $\sigma_{y}^{2}$ are the variance of x and y, $\sigma_{xy}$ is the covariance of x and y, $c_{1}=0.0001$ and $c_{2}=0.0009$ are set due to the image intensity range of 1.

(Equation 11) $$\text{LPIPS}=\sum_{l}{\left\|F_{l}\left(X^{\prime }\right)-F_{l}\left(X\right)\right\|}_{2}^{2}$$

where F is the pre-trained AlexNet,^49^ $F_{l}\left(\cdot \right)$ indicates the features of the l th layer of F.

(Equation 12) $$\text{DSC}=\frac{2\left|M^{\prime }\cap M\right|}{\left|M^{\prime }\right|+\left|M\right|}$$

where |·| is the cardinality of the set.

(Equation 13) $$\text{ASSD}=\frac{\sum_{x\in \partial M}d\left(x,\partial M^{\prime }\right)+\sum_{y\in \partial M^{\prime }}d\left(y,\partial M\right)}{\left|\partial M^{\prime }\right|+\left|\partial M\right|}$$

where $\partial M^{\prime }$ and ∂M are the contour sets of $M^{\prime }$ and M, d(·,·) indicates the minimum distance between a point and a contour.

(Equation 14) $$\text{AUC}=\frac{\sum_{ins_{i}\in pos}\mathrm{ran}k_{ins_{i}}-\frac{P\times \left(P+1\right)}{2}}{P\times N}$$

where P and N are the number of positive and negative samples. $\mathrm{ran}k_{ins_{i}}$ refers to the serial number of sample i. $\sum_{ins_{i}\in pos}\mathrm{ran}k_{ins_{i}}$ indicates the sum of serial numbers of the positive samples.

## Supplemental information

Document S1. Figures S1–S7 and Table S1–S7

Document S2. Article plus supplemental information
