# Supplementary material for: All-in-one medical image-to-image translation
Source: Cell Rep Methods. 2025 Aug 11;5(8):101138. doi: 10.1016/j.crmeth.2025.101138 (PMC12461644; doi:10.1016/j.crmeth.2025.101138)
Supplement: Document S1. Figures S1–S7 and Table S1–S7 [file mmc1.pdf]

Cell Reports Methods, Volume *5*

Supplemental information

All-in-one medical image-to-image translation

Luyi Han, Tao Tan, Yunzhi Huang, Haoran Dou, Tianyu Zhang, Yuan Gao, Xin Wang, Chunyao Lu, Xinglong Liang, Yue Sun, Jonas Teuwen, S. Kevin Zhou, and Ritse Mann

Supplementary Figures

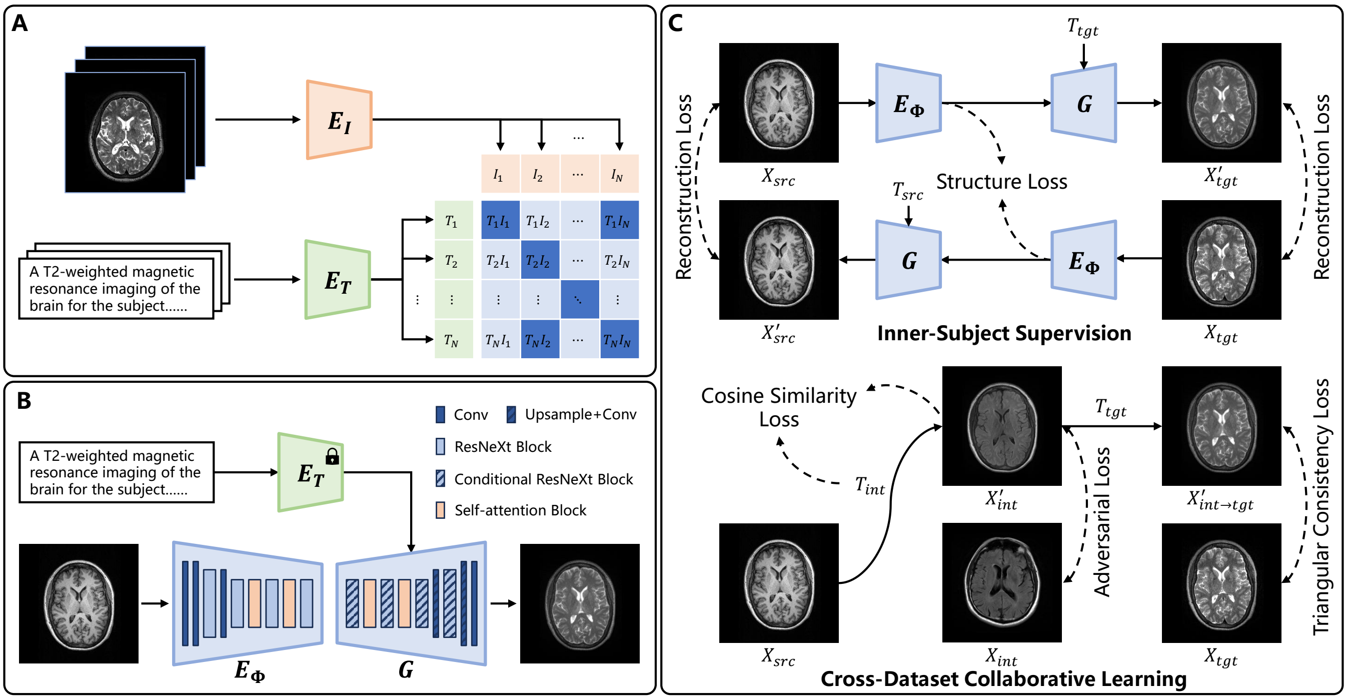

Figure S1: **The overview of the proposed framework, related to Figure 1.** (A) DCLIP jointly trains the encoders by predicting the correct pairings for a batch of <image, prompt> examples. Note that, one prompt may correspond to multiple images. (B) The architecture of the DCLIP-driven synthesis model. (C) The training procedure of inner-subject supervision (top) and cross-dataset collaborative learning (bottom).

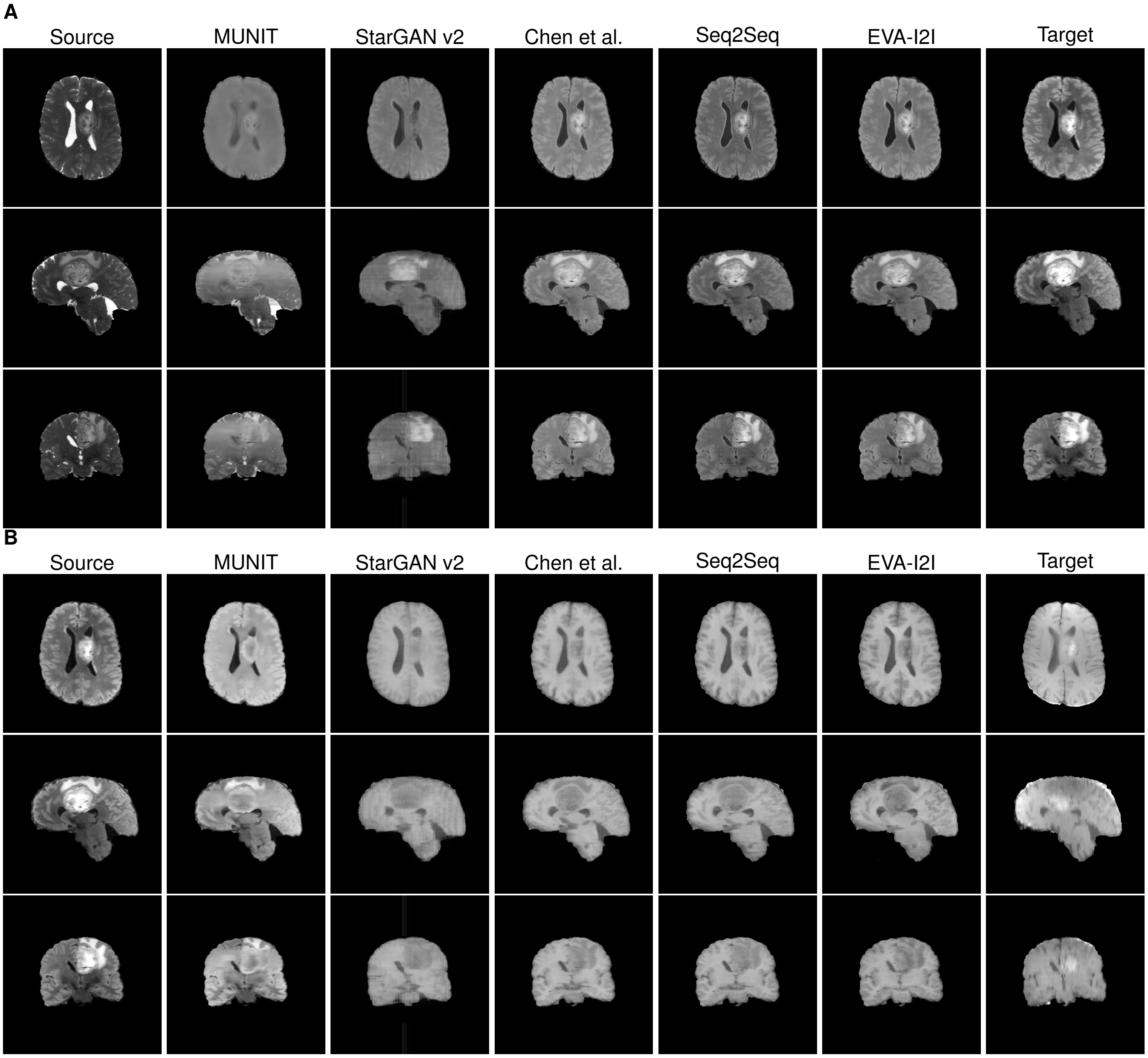

Figure S2: **Visualization of image-to-image translation in BraTS2021 by comparison methods, related to Figure 2.** (A) Visualization of transferring T2 to Flair. (B) Visualization of transferring Flair to T1.

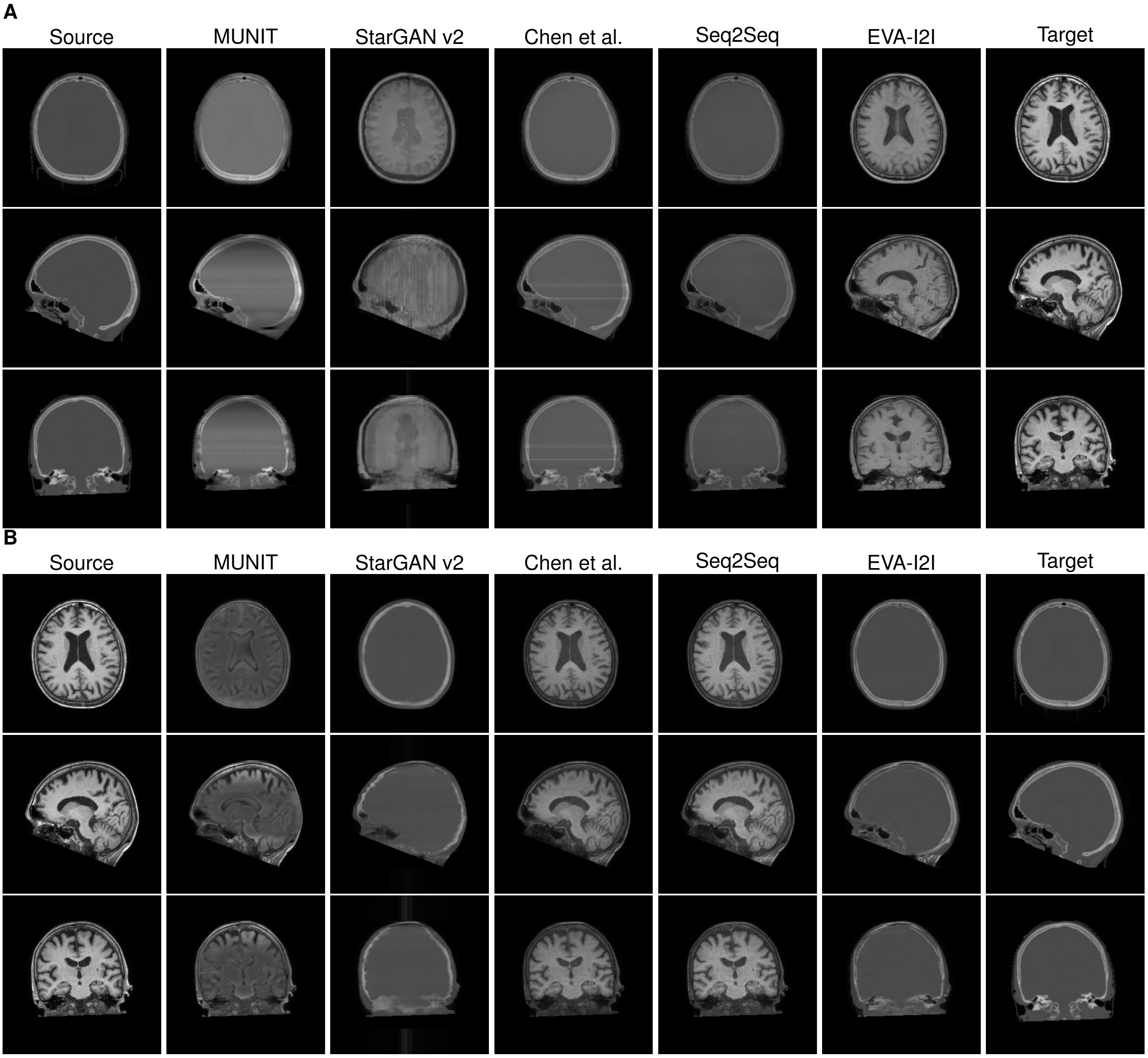

Figure S3: **Visualization of image-to-image translation in SynthRAD2023 by comparison methods, related to Figure 2.** (A) Visualization of transferring CT to T1. (B) Visualization of transferring T1 to CT.

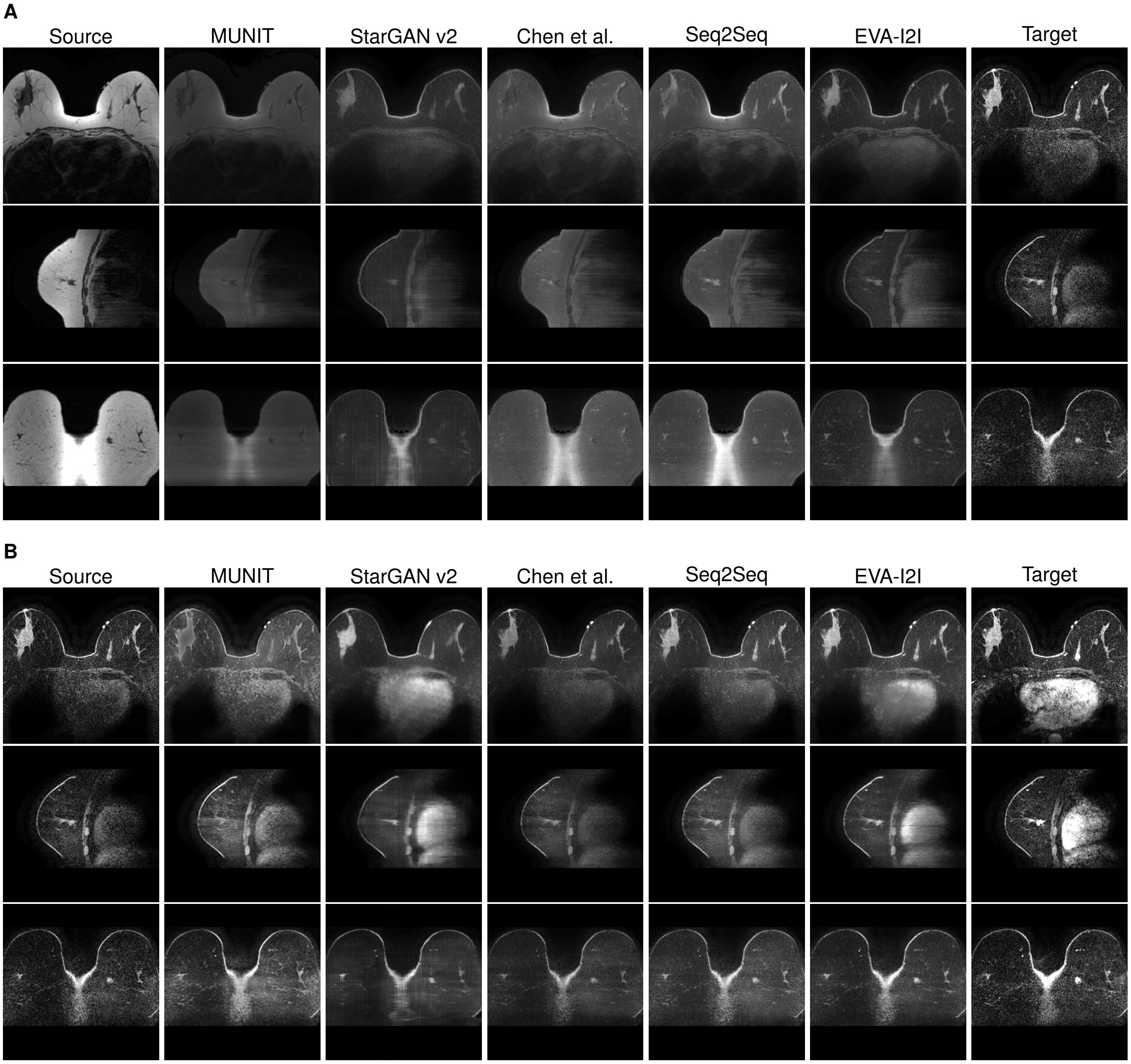

Figure S4: **Visualization of image-to-image translation in Duke Breast Cancer MRI Dataset by comparison methods, related to Figure 2.** (A) Visualization of transferring non-fat-saturated T1 to fat-saturated T1. (B) Visualization of transferring pre-contrast DCE to post-contrast DCE.

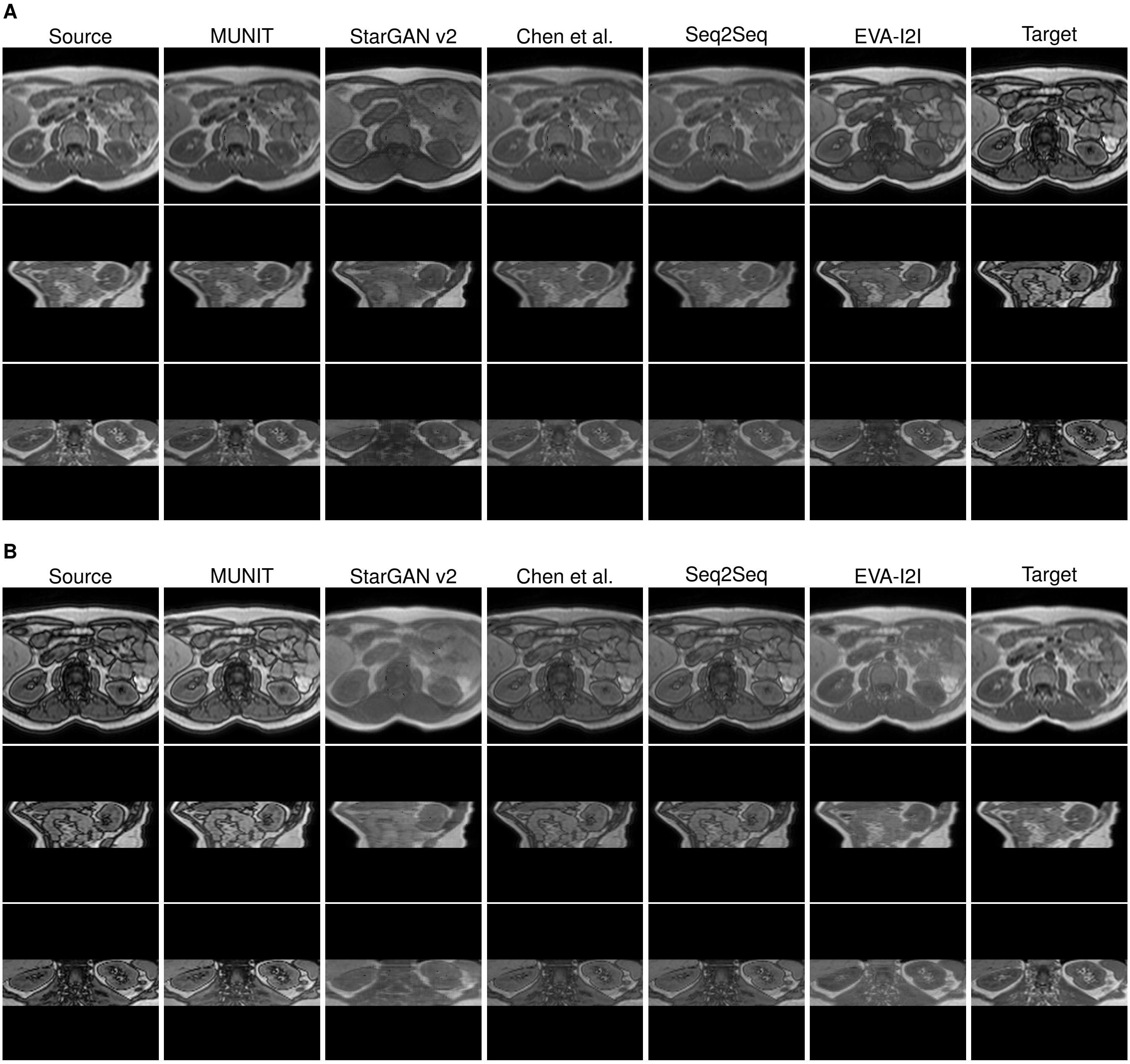

Figure S5: **Visualization of image-to-image translation in CHAOS by comparison methods, related to Figure 2.** (A) Visualization of transferring IP to OOP. (B) Visualization of transferring OOP to IP.

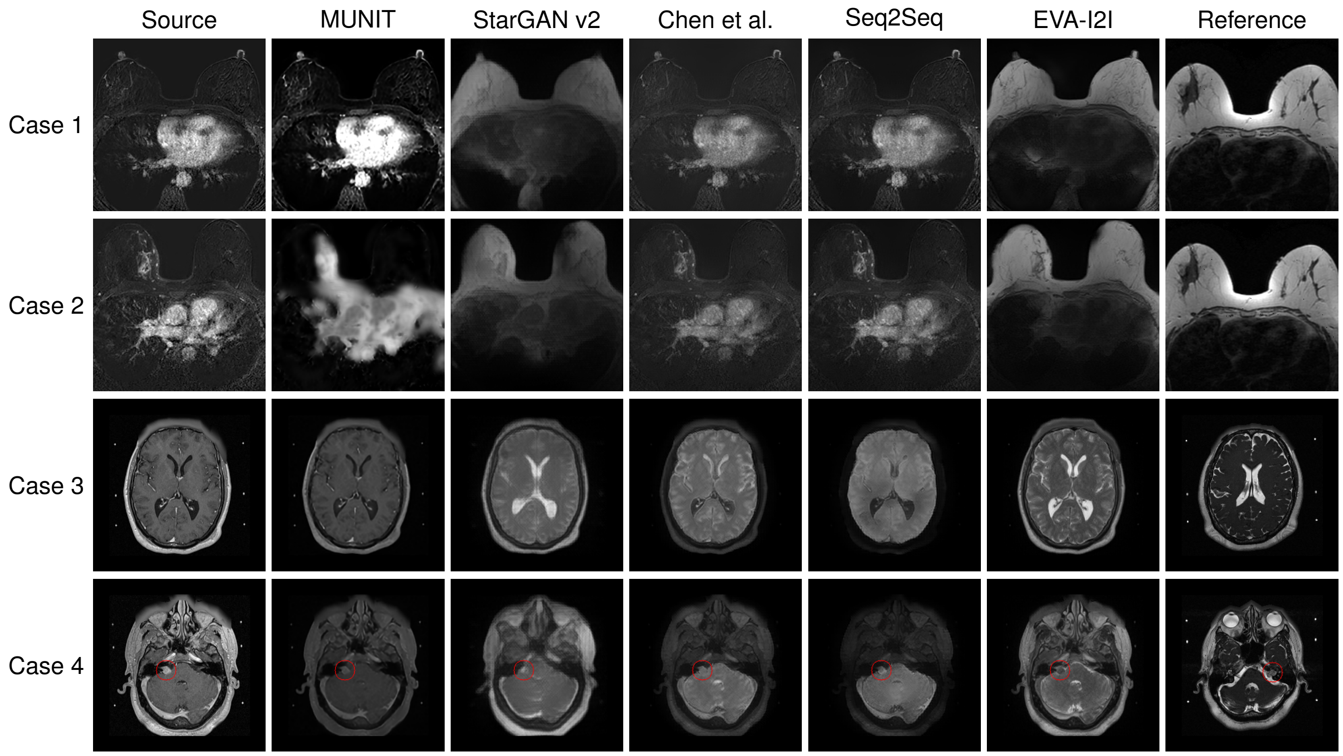

Figure S6: **Axial visualization of zero-shot I2I translation for never-before-seen domains by comparison methods, related to Figure 2.** Case 1 and case 2 show the transformation from wash-in (subtraction between post- and pre-contrast DCE) to T1 image in the in-house dataset. Case 3 and case 4 show the transformation from T1Gd (with fat-saturation and remaining skulls) to T2 image in the crossMoDA22 dataset. case 3 exhibits normal brain regions, whereas case 4 depicts instances of vestibular schwannoma (in red circles). They are both zero-shot DA tasks because the wash-in images in the in-house dataset and T1Gd images in crossMoDA22 are never-before-seen domains in the in-domain datasets. Note that, the reference image is from a different subject because the input case lacks the corresponding image of the reference prompt.

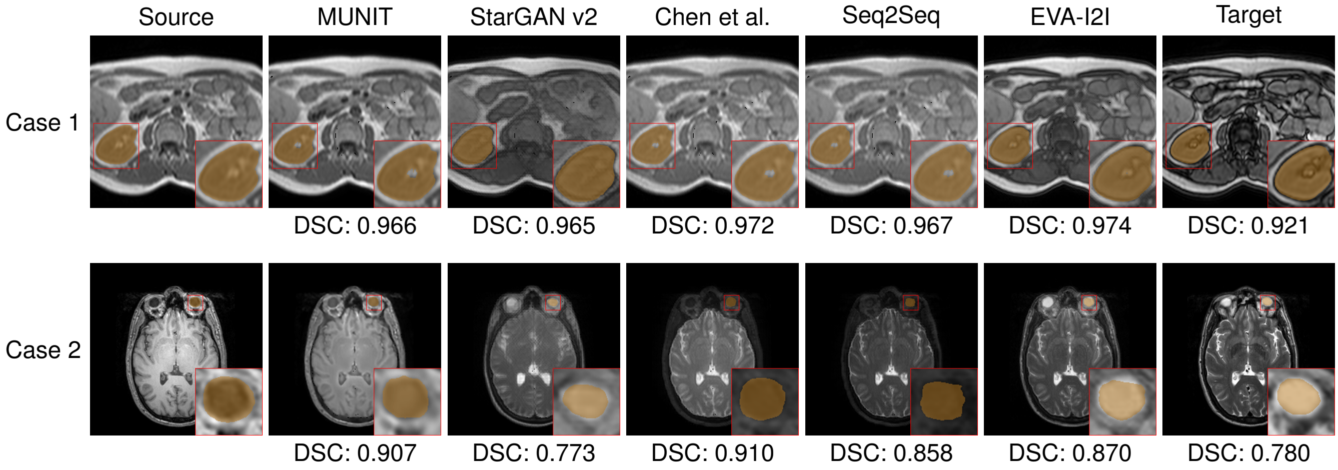

Figure S7: **Axial visualization of segmentation based on MedSAM, related to Figure 2.** We compare the segmentation masks derived from source images and their corresponding translated or target images. The translated images achieved higher DSC scores than the target images, which may be due to imperfect alignment between the source and target images. Furthermore, although MUNIT and Chen et al. achieve higher DSC in Case 2, they fail to translate the image into the desired target domain accurately.

Supplementary Tables

Table S1: **The detailed information for in- and out-domain datasets, related to Figure 1.** In-domain datasets (D01-D07) are used to build the proposed EVA-I2I. Out-domain datasets (D08-D12) are used for external validation and downstream applications.

Datasets	Organ	Sequence/Modality	Subjects	Scans (3D volumes)
D01. AMOS22	Abdomen	T1, CT	600	600
D02. BraTS2021	Brain	T1, T1Gd, T2, Flair	1,470	5,880
D03. CHAOS	Abdomen	IP, OOP, T2, CT	80	160
D04. Duke Breast Cancer MRI Dataset	Breast	T1, DCE	922	5,033
D05. IXI	Brain	T1, T2, PD, MRA	581	2,304
D06. SynthRAD2023	Brain, Pelvis	T1, CT, CBCT	720	1,440
D07. OASIS-3	Brain	T1, T2, T2*, TSE-T2, Flair, SWI	4,104	12,533
D08. crossMoDA22	Brain	T1Gd, T2	618	618
D09. Learn2Reg-OASIS	Brain	T1	414	414
D10. LEMON	Brain	T1, T2	226	452
D11. HCP Retest	Brain	T1, T2	45	90
D12. In-house Dataset	Breast	T1, T2, DCE, DWI	2,101	56,832

Table S2: **The detailed information of DICOM tags used for building medical prompts, related to Figure 1.**

Tag	Keyword
(0008,0070)	Manufacturer
(0008,1090)	ManufacturerModelName
(0008,0032)	AcquisitionTime
(0018,0020)	ScanningSequence
(0018,0021)	SequenceVariant
(0018,0022)	ScanOptions
(0018,0080)	RepetitionTime
(0018,0081)	EchoTime
(0018,0082)	InversionTime
(0018,0087)	MagneticFieldStrength
(0018,1314)	FlipAngle
(0018,0060)	KVP
(0018,1150)	ExposureTime
(0018,1151)	XRayTubeCurrent

Table S3: **The quantitative results of image-to-image translation on in-domain datasets, related to Table 1.** The best result is in bold, and the second best one is underlined.

Dataset	Method	PSNR ↑	SSIM ↑	LPIPS ↓
BraTS2021	MUNIT	24.9±2.2	0.929±0.020	12.60±3.15
	StarGAN v2	25.3±1.6	0.911±0.010	16.16±2.31
	Chen et al.	26.3±2.2	<u>0.950±0.012</u>	7.62±2.53
	Seq2Seq	<u>26.3±2.1</u>	0.946±0.918	<u>7.56±2.44</u>
	EVA-I2I	**26.8±2.1**	**0.957±0.012**	**6.58±2.32**
IXI	MUNIT	22.4±2.8	<u>0.876±0.064</u>	<u>22.9±12.7</u>
	StarGAN v2	<u>23.1±3.3</u>	0.841±0.091	27.9±17.5
	Chen et al.	21.5±3.5	0.856±0.051	26.4±16.4
	Seq2Seq	21.2±4.0	0.849±0.070	25.9±17.9
	EVA-I2I	**24.6±4.0**	**0.890±0.085**	**17.1±16.2**
OASIS-3	MUNIT	21.8±2.7	**0.841±0.083**	<u>26.9±13.0</u>
	StarGAN v2	<u>22.3±2.6</u>	<u>0.794±0.118</u>	32.0±15.1
	Chen et al.	20.1±2.6	0.789±0.056	32.4±15.3
	Seq2Seq	20.7±2.7	0.782±0.075	30.3±15.7
	EVA-I2I	**23.1±3.5**	0.793±0.152	**23.1±15.8**
SynthRAD2023	MUNIT	22.7±4.9	<u>0.834±0.100</u>	32.4±17.7
	StarGAN v2	<u>23.9±4.4</u>	**0.836±0.070**	<u>28.7±11.8</u>
	Chen et al.	20.3±5.3	0.797±0.099	38.0±21.3
	Seq2Seq	20.5±5.1	0.805±0.095	37.2±20.7
	EVA-I2I	**23.9±4.4**	0.802±0.114	**22.0±11.4**
Duke Breast Cancer MRI Dataset	MUNIT	<u>20.6±3.2</u>	**0.677±0.102**	52.3±17.4
	StarGAN v2	20.4±2.2	0.626±0.073	50.4±11.4
	Chen et al.	19.1±3.1	<u>0.654±0.106</u>	<u>38.1±17.8</u>
	Seq2Seq	19.3±2.5	0.642±0.096	39.5±15.7
	EVA-I2I	**21.9±2.4**	0.648±0.085	**28.1±12.8**
CHAOS	MUNIT	18.5±2.4	<u>0.760±0.064</u>	33.4±10.4
	StarGAN v2	<u>20.0±2.2</u>	**0.767±0.056**	40.9±9.9
	Chen et al.	16.5±2.5	0.748±0.070	<u>31.9±9.4</u>
	Seq2Seq	17.3±2.0	0.750±0.066	33.9±10.6
	EVA-I2I	**20.5±2.8**	0.730±0.130	**31.2±13.7**

Table S4: **The quantitative results of image synthesis on LEMON (out-domain) dataset for compared models trained with single dataset and multiple datasets, related to Table 1.** The best result is in bold, and the second best one is underlined.

Dataset	Method	T1→T2			T2→T1		
		PSNR (dB) ↑	SSIM ↑	LPIPS ↓	PSNR (dB) ↑	SSIM ↑	LPIPS ↓
Single	Pix2Pix (BraTS2021)	22.7±0.7	0.902±0.011	11.2±1.2	24.9±1.0	0.928±0.012	10.5±1.2
	Pix2Pix (IXI)	21.7±0.9	0.903±0.009	11.0±0.9	24.0±0.7	0.922±0.010	13.9±1.2
	Pix2Pix (OASIS-3)	20.9±0.7	0.900±0.010	11.2±1.0	24.1±0.8	0.924±0.010	12.8±1.2
Multiple	MUNIT	17.6±0.6	0.875±0.005	17.6±1.3	23.4±0.7	0.892±0.009	18.2±1.9
	StarGAN v2	19.7±0.6	0.873±0.005	21.0±1.1	23.9±0.9	0.899±0.008	18.9±1.7
	Chen et al.	21.9±0.6	0.916±0.010	11.6±1.2	24.3±1.0	**0.934±0.012**	10.7±1.3
	Seq2Seq	20.5±0.6	0.859±0.010	12.3±1.2	24.7±1.1	0.931±0.013	10.1±1.2
	EVA-I2I	**22.8±0.7**	**0.919±0.011**	**10.7±1.2**	**25.3±1.1**	0.933±0.012	**9.29±1.16**

Table S5: **The quantitative results of image-to-image translation on HCP Retest (out-domain) dataset for compared models trained with single dataset and multiple datasets, related to Table 1.** The best result is in bold, and the second best one is underlined.

Dataset	Method	T1→T2			T2→T1		
		PSNR ↑	SSIM ↑	LPIPS ↓	PSNR ↑	SSIM ↑	LPIPS ↓
Single	Pix2Pix (BraTS2021)	<u>23.7±0.6</u>	0.927±0.006	7.63±0.58	<u>27.0±1.5</u>	0.956±0.010	<u>7.30±1.26</u>
	Pix2Pix (IXI)	22.3±0.5	0.923±0.005	9.73±0.90	25.1±0.4	0.944±0.006	10.42±0.53
	Pix2Pix (OASIS-3)	21.8±0.5	0.920±0.005	9.52±0.83	24.4±0.8	0.932±0.007	9.84±0.97
Multiple	MUNIT	18.7±0.6	0.857±0.008	14.14±1.22	21.9±0.5	0.868±0.009	13.54±1.08
	StarGAN v2	21.2±0.7	0.887±0.005	16.91±0.69	23.8±1.0	0.901±0.008	19.80±1.16
	Chen et al.	23.6±0.6	**0.949±0.006**	<u>7.54±0.57</u>	25.0±1.2	0.956±0.010	9.06±1.25
	Seq2Seq	21.4±0.5	0.881±0.007	8.41±0.60	26.0±1.4	<u>0.958±0.010</u>	7.93±1.28
	EVA-I2I	**24.2±0.6**	<u>0.948±0.007</u>	**7.38±0.55**	**27.3±1.5**	**0.963±0.010**	**6.82±1.16**

Table S6: **The DSC results of T1-to-T1 mono-modality (Lear2Reg-OASIS) and T2-to-T1 cross-modality (HCP Retest and LEMON) registration for brain MRI, related to Figure 5.** The best result is in bold.

Method	T1→T1	T2→T1	
	Learn2Reg ↑	HCP Retest ↑	LEMON ↑
Rigid	0.574±0.020	0.577±0.023	0.545±0.010
VM	**0.837±0.010**	0.449±0.024	0.407±0.025
+ IMT	-	0.627±0.017	0.633±0.020
+ MUNIT	-	0.537±0.025	0.572±0.024
+ StarGAN v2	-	0.632±0.023	0.611±0.016
+ Chen et al.	-	0.697±0.025	0.648±0.023
+ Seq2Seq	-	0.699±0.023	0.654±0.024
+ EVA-I2I	-	**0.701±0.022**	**0.655±0.024**

Table S7: **The quantitative results of vestibular schwannoma (VS) segmentation on crossMoDA22 dataset (T1Gd-to-T2 cross-modality segmentation), related to Figure 5.** The best zero-shot DA result is in bold, and the second best one is underlined.

Method	Small VS (<1 cm)		Medium VS (1-2 cm)		Large VS (>2 cm)	
	DSC ↑	ASSD ↓	DSC ↑	ASSD ↓	DSC ↑	ASSD ↓
MSF-Net	0.774±0.055	0.441±0.087	0.871±0.051	0.518±0.216	0.867±0.086	0.819±0.388
nnU-Net	0.000±0.000	45.3±28.4	0.332±0.371	28.5±33.0	0.512±0.300	25.3±40.0
+ MUNIT	0.000±0.000	47.8±27.7	0.048±0.180	47.1±32.8	0.110±0.191	52.2±35.3
+ StarGAN v2	0.100±0.202	28.2±18.7	0.417±0.306	7.98±11.55	0.305±0.192	7.98±7.56
+ Chen et al.	0.205±0.235	16.6±22.4	<u>0.542±0.293</u>	<u>6.84±13.06</u>	<u>0.692±0.106</u>	<u>1.78±0.59</u>
+ Seq2Seq	<u>0.317±0.265</u>	**16.3±23.5**	0.373±0.322	10.1±16.2	0.438±0.299	3.57±2.22
+ EVA-I2I	**0.349±0.294**	<u>16.4±23.9</u>	**0.701±0.279**	**4.54±16.68**	**0.866±0.048**	**0.779±0.245**
